# Glutathione S-transferases play a role in the detoxification of flumethrin and chlorpyrifos in *Haemaphysalis longicornis*

**DOI:** 10.1186/s13071-018-3044-9

**Published:** 2018-08-09

**Authors:** Emmanuel Pacia Hernandez, Kodai Kusakisako, Melbourne Rio Talactac, Remil Linggatong Galay, Takeshi Hatta, Kozo Fujisaki, Naotoshi Tsuji, Tetsuya Tanaka

**Affiliations:** 1 Laboratory of Infectious Diseases, Joint Faculty of Veterinary Medicine, Kagoshima University, 1-21-24 Korimoto, Kagoshima, 890-0056 Japan; 2 Department of Pathological and Preventive Veterinary Science, The United Graduate School of Veterinary Science, Yamaguchi University, Yoshida, Yamaguchi, 753-8515 Japan; 3 Department of Clinical and Population Health, College of Veterinary Medicine and Biomedical Sciences, Cavite State University, 4122 Cavite, Philippines; 4 Department of Veterinary Paraclinical Sciences, University of the Philippines Los Baños, College, 3004 Laguna, Philippines; 5 Department of Parasitology, Kitasato University School of Medicine, Kitasato, Minami, Sagamihara, Kanagawa 252-0374 Japan; 6 National Agricultural and Food Research Organization, 3-1-5 Kannondai, Tsukuba, Ibaraki 305-0856 Japan

**Keywords:** *Haemaphysalis longicornis*, Glutathione S-transferase, Acaricides, Flumethrin, Chlorpyrifos, Tick

## Abstract

**Background:**

*Haemaphysalis longicornis* is a tick of importance to health, as it serves as a vector of several pathogens, including *Theileria orientalis*, *Babesia ovata*, *Rickettsia japonica* and the severe fever with thrombocytopenia syndrome virus (SFTSV). Presently, the major method of control for this tick is the use of chemical acaricides. The glutathione S-transferase (GST) system is one mechanism through which the tick metabolizes these acaricides. Two GSTs from *H. longicornis* (HlGST and HlGST2) have been previously identified.

**Results:**

Enzyme kinetic studies were performed to determine the interaction of acaricides with recombinant *H. longicornis* GSTs. Recombinant HlGST activity was inhibited by flumethrin and cypermethrin, while recombinant HlGST2 activity was inhibited by chlorpyrifos and cypermethrin. Using real-time RT-PCR, the upregulation of the *HlGST* gene was observed upon exposure to sublethal doses of flumethrin, while the *HlGST2* gene was upregulated when exposed to sublethal doses of chlorpyrifos. Sex and strain dependencies in the induction of *GST* gene expression by flumethrin were also observed. Knockdown of the *HlGST* gene resulted in the increased susceptibility of larvae and adult male ticks to sublethal doses of flumethrin and the susceptibility of larvae against sublethal doses of chlorpyrifos was increased upon knockdown of *HlGST2*.

**Conclusions:**

HlGST could be vital for the metabolism of flumethrin in larvae and adult male ticks, while HlGST2 is important in the detoxification of chlorpyrifos in larval ticks.

**Electronic supplementary material:**

The online version of this article (10.1186/s13071-018-3044-9) contains supplementary material, which is available to authorized users.

## Background

The hard tick *Haemaphysalis longicornis* is a blood-sucking arthropod widely distributed in East Asia and Australia. They are known vectors of *Theileria orientalis*, *Babesia ovata* and *Rickettsia japonica*, as well as the severe fever with thrombocytopenia syndrome virus (SFTSV) [1, 2]. Until now, tick control has relied mainly upon the application of acaricides such as amitraz, synthetic pyrethroids and organophosphates [3, 4]. The continuous use of acaricides has some ill effects, such as the development of resistance against these acaricides [5]. One factor that has contributed to the development of acaricide resistance is the improper application of acaricides, with a particular emphasis on sublethal doses [6].

Amitraz is a formamidine acaricide that has been around since the 1960s. It acts as an agonist of the octopaminergic receptors of arthropods, leading to the stimulation of monoamine oxidases and G proteins. This stimulation leads to the synthesis of cAMP and cGMP, and could alter the behavior of the arthropods [7]. On the other hand, organophosphates are acetylcholinesterase inhibitors. Their action results in acetylcholine continuous stimulation, causing hyperactivity and, eventually, the death of the arthropods. Organophosphates possess a broad spectrum of activity against insects and acarians [7]. Pyrethroids are esters capable of opening Na^+^ channels, resulting in the depolarization of nerve cell membranes. The effect on arthropods involves two phases. The first phase is the reversible “knockdown” effect, wherein arthropods cease all movements and act as if they are dead. This is caused by pyrethroids acting on the cerebral ganglia. Arthropods may still wake-up, and then go to the second phase, wherein the pyrethroids may affect peripheral nerves. This could result in brief, rapid and inconsistent movements of arthropods, perhaps leading eventually to death. The pyrethroids permethrin, cypermethrin and deltamethrin are both acaricides and insecticides, while flumethrin is mainly an acaricide [7].

Until now, the exact mechanisms of acaricide metabolism by the ticks are not fully understood. Three metabolic pathways are believed to play roles in this detoxification process: carboxylesterases, monooxygenases and glutathione S-transferases [6]. Glutathione S-transferases (GSTs) are multifunctional enzymes that are responsible for the metabolism and detoxification of both xenobiotic and physiological substances. Metabolism involves the catalysis of thiol additions of the reduced glutathione to organic compounds through their electrophilic centers. The formation of more water-soluble conjugates would facilitate their elimination [8]. The transport of molecules is facilitated by an ATP-requiring active transport system through the multidrug resistance-related protein (MRP) [6].

We have already identified two GST molecules of *H. longicornis* [9]. GSTs act on a wide variety of substrates, and each GST isoenzyme may function very differently from the others, wherein not all GSTs are involved in the detoxification of acaricides [10, 11]. Therefore, it is important to determine the role of the GSTs of ticks in acaricide metabolism. Targeting specific GSTs that impede the ability of the arthropod to survive acaricide application could be included in the development of a tick control plan [12]. In this study, we determined the possibility of the interaction of recombinant GSTs and several acaricides. We also observed the ability of sublethal doses of acaricides to induce GST gene and protein expression. Finally, we were able to establish the significance of GSTs in the metabolism of sublethal doses of acaricides through RNA interference (RNAi) experiments.

## Methods

### Ticks and experimental animals

The parthenogenetic Okayama strain and the bisexual Oita strain of *H. longicornis* were used in the experiments throughout this study. In Japan, no evidence of resistance against acaricides was reported in this tick species. Ticks were maintained by feeding on the ears of Japanese white rabbits (KBT Oriental, Saga, Japan) for several generations at the Laboratory of Infectious Diseases, Joint Faculty of Veterinary Medicine, Kagoshima University, Kagoshima, Japan [13]. Experimental animals were kept at 25 °C and 40% relative humidity, with a constant supply of water and commercial feeds. The ticks, on the other hand were kept in glass tubes sealed with cotton plug and maintained at 15 °C and 80–85% relative humidity in an incubator. The ticks were maintained for 2 to 3 months after hatching or molting before use. The care and use of experimental animals in this study were approved by the Animal Care and Use Committee of Kagoshima University (Approval number VM15055).

### Chemicals

Organophosphate acaricides (ethion, coumaphos, chlorpyrifos and diazinon), pyrethroids (cypermethrin and flumethrin), an avermectin (ivermectin), and a formamidine compound (amitraz) were evaluated for their interaction with the GSTs of *H. longicornis*. Ethion, chlorpyrifos, diazinon, cypermethrin and amitraz were purchased from Wako Pure Chemical Industries, Ltd. (Osaka, Japan). Coumaphos, ivermectin and flumethrin were purchased from Sigma-Aldrich (St. Louis, MO, USA).

### Enzyme activity assay

The inhibition activity of recombinant GSTs was measured according to the methods of Habig and da Silva [3, 14], using 1-chloro-2,4-dinitrobenzene (CDNB) (Sigma-Aldrich) as a substrate. The recombinant GSTs used in this study were expressed as described previously [9]. Two-hundred microliters of the reaction mixture consisting of varying concentrations of CDNB (0.125, 0.25, 0.5, 1 and 2 mM) dissolved in methanol, 5 mM glutathione, 0.1 mM of acaricide dissolved in methanol, and 120 μM recombinant GST in 100 mM Tris-HCl (pH 7.5) or without recombinant GST for the blank was tested in a 96-well plate. The methanol concentration was maintained at 5%. Equine liver GST and recombinant *H. longicornis* 2-cys-peroxiredoxin [15] were used as the positive and negative controls, respectively. Absorbance (A_340nm_) was measured each minute in an SH-9000 microplate reader (Corona Electric, Ibaraki, Japan) at 25 °C for 5 min. The extinction coefficient of 9.6 mM^-1^cm^-1^, corrected for the 96-well microplate light path, was used. Each assay was done in triplicate, and the results were expressed as the mean of three separate experiments. Kinetic constants *K*_*m*_ and *V*_*max*_ were calculated from the double-reciprocal plot of 1/v *versus* the 1/[S] or Lineweaver-Burk plot in which *V*_*max*_ = 1/y-intercept of the regression line and *K*_*m*_ = *V*_*max*_ × slope of the regression line.

### Determination of acaricide sublethal dose

The following acaricides were dissolved in methanol at 4 dilutions: flumethrin (0.4 μM, 4 μM, 40 μM and 400 μM), chlorpyrifos (0.01 mM, 0.1 mM, 1 mM and 10 mM), and amitraz (0.01 mM, 0.1 mM, 1 mM and 10 mM). These concentrations were based on previous studies [6, 16].

For exposure studies, the methods of Duscher [6] were used with some modifications. Briefly, 0.5 ml of each dilution was spotted onto a 10 × 5 cm piece of filter paper in scattered dots and dried under the fume hood for at least 2 h. Each group of 10 parthenogenetic females, nymphs and larvae, as well as bisexual male and female ticks, was placed in the acaricide-impregnated filter paper and exposed for 48 h. Mortality was checked after 48 h. For the parthenogenetic larvae and nymphs, further 10-fold dilutions were made to determine the sublethal dose. The maximum sublethal dose in this experiment is the highest dose that has either failed to cause any mortality or caused just a single mortality out of all the ticks tested [6].

### GST gene and protein expression analysis of parthenogenetic female ticks exposed to flumethrin, chlorpyrifos and amitraz

Parthenogenetic female ticks were exposed to different sublethal concentrations of flumethrin (0, 0.4, 4 and 40 μM), chlorpyrifos (0, 0.01, 0.1 and 1 mM), and amitraz (0, 0.01, 0.1 and 1 mM). Total RNA was extracted from whole tick samples by homogenizing using an automill (Tokken, Chiba, Japan) and were added to TRI Reagent® (Sigma-Aldrich). RNA extraction was performed following the manufacturer’s protocol. Subsequently, single-stranded cDNA was prepared by reverse transcription using the ReverTra Ace® cDNA Synthesis Kit (Toyobo, Osaka, Japan), following the manufacturer’s protocol. Transcription analysis of *HlGST* and *HlGST2* genes was performed through real-time RT-PCR using THUNDERBIRD™ SYBR® qPCR Mix (Toyobo) with an Applied Biosystems 7300 Real-Time PCR System using HlGST and HlGST2 real-time gene-specific primers (Table 1). Standard curves were made from fourfold serial dilutions of the cDNA of adult parthenogenetic ticks fed for 3 days. The PCR cycle profile used is as follows: 95 °C for 10 min, 40 cycles of a denaturation step at 95 °C for 15 s, and an annealing/extension step at 60 °C for 60 s. The data were analyzed with Applied Biosystems 7300 system SDS software. *Actin*, *tubulin*, *P0* and *L23* genes were evaluated for standardization at the first step of real-time RT-PCR. *P0* genes were selected as an internal control for the ticks.

**Table 1:** Gene-specific primers used in this study. Italics denotes RNA polymerase promoter sequences

Primer	Sequence [5'→3']
HlGST real-time forward	CTTCTTGGATCTTGGCGGGT
HlGST real-time reverse	CGATGTCCCAGTAGCCGAG
HlGST RT forward	ACGTGAAGCTCACCCAGAGCAT
HlGST RT reverse	AAGCTAGCCATGTCGCCGTTGA
HlGST RNAi forward	GCCTGGCTCAAGGAGAAACACA
HlGST RNAi reverse	ACAAAGGCCTTCAGGTTGGGGA
HlGST T7 forward	*TAATACGACTCACTATAGG*GCCTGGCTCAAGGAGAAACACA
HlGST T7 reverse	*TAATACGACTCACTATAGG*ACAAAGGCCTTCAGGTTGGGGA
HlGST2 real-time forward	CCCTTCCGGGAATGAAGGAG
HlGST2 real-time reverse	GATCGCTCAGCAGTCGTCAG
HlGST2 RT forward	ACGTCAAGCTGACGCAGAGCAT
HlGST2 RT reverse	ATGGGCCAAGCCTTGAAGCGAT
HlGST2 RNAi forward	AGGATAAAAGGTACGGCTTCGGCA
HlGST2 RNAi reverse	TTTCACGATCTGGAGAGCCTCGTA
HlGST2 T7 forward	*TAATACGACTCACTATAGG*AGGATAAAAGGTACGGCTTCGGCA
HlGST2 T7 reverse	*TAATACGACTCACTATAGG*TTTCACGATCTGGAGAGCCTCGTA
P0 real-time forward	CTCCATTGTCAACGGTCTCA
P0 real-time reverse	TCAGCCTCCTTGAAGGTGAT
L23 real-time forward	CACACTCGTGTTCATCGTCC
L23 real-time reverse	ATGAGTGTGTTCACGTTGGC
Actin real-time forward	ATCCTGCGTCTCGACTTGG
Actin real-time reverse	GCCGTGGTGGTGAAAGAGTAG
Actin RT forward	CCAACAGGGAGAAGATGACG
Actin RT reverse	ACAGGTCCTTACGGATGTCC
Tubulin real-time forward	TTCAGGGGCCGTATGAGTAT
Tubulin real-time reverse	TGTTGCAGACATCTTGAGGC
EGFP T7 forward	*TAATACGACTCACTATAGG*GACGTAAACGGCCACAAGTT
EGFP T7 reverse	*TAATACGACTCACTATAGG*TGCTCAGGTAGTGGTTGTCG

The protein was also extracted from the abovementioned parthenogenetic female ticks exposed to flumethrin, chlorpyrifos and amitraz. Whole tick samples were also homogenized using an automill (Tokken), and then suspended in phosphate-buffered saline (PBS) treated with Complete Mini Protease Inhibitor Cocktail Tablets (Roche, Mannheim, Germany). After sonication and recovery of the supernatant, tick proteins were separated with 12% SDS-polyacrylamide gel electrophoresis (SDS-PAGE) and transferred to a polyvinylidene difluoride (PVDF) membrane (Millipore, Billerica, MA, USA). The membrane was blocked overnight with 3% skim milk in PBS with 0.05% Tween 20, and it was then incubated with a primary antibody using mouse anti-GST sera (1:1000 dilution) for 1 h. β-tubulin was used as a control [17]. After incubation with horseradish peroxidase-conjugated goat anti-mouse IgG (1:50,000 dilution; DakoCytomation, Glostrup, Denmark) for 1 h, the signal was detected using Clarity™ Western ECL Substrate (Bio-Rad Laboratories, Hercules, CA, USA) and analyzed using FluorChem FC2 software (Alpha Innotech, San Leandro, CA, USA).

### RNA interference

RNA interference using double-stranded RNA (dsRNA) was performed to determine the effect of GST on survival upon acaricide exposure. The PCR primers used for the synthesis of dsRNA are listed in Table 1. The *HlGST* and *HlGST2* fragments were amplified by PCR from plasmid clones using oligonucleotides, including HlGST T7 forward with HlGST RNAi reverse and HlGST T7 reverse with HlGST RNAi forward primers, and HlGST2 T7 forward with HlGST2 RNAi reverse and HlGST2 T7 reverse with HlGST2 RNAi forward primers, to attach the T7 promoter recognition sites on both forward and reverse ends. *Enhanced green fluorescent protein* (*EGFP*) gene fragments were amplified from *pEGFP* through PCR using oligonucleotides containing EGFP T7 forward and EGFP T7 reverse primers as well. PCR products were purified using a GENECLEAN II Kit (MP Biomedicals, Ilkrich, France). The T7 RiboMAX Express RNAi System (Promega, Madison, WI, USA) was used to synthesize dsRNA by *in vitro* transcription. The successful construction of dsRNA was confirmed by running 1 μl of the dsRNA products in 1.5% agarose gel in a Tris-acetate-EDTA (TAE) buffer. *HlGST*, *HlGST2*, or *HlGST 1/2* dsRNA (0.5 μl) dissolved in high purity water was injected to the hemocoel of unfed adult ticks through the fourth coxa at 1μg/tick concentration [9]. A total of 35 ticks per group were injected with dsRNA. The control group was injected with *EGFP* dsRNA. After injection, the ticks were held for 24 h in a 25 °C incubator to check for mortality resulting from injury during injection. The ticks were then kept in vials sealed with cotton plug, placed in a glass chamber, and maintained at 25 °C and 80–85% relative humidity in an incubator for another 72 h. For larvae and nymphs, the dsRNA immersion method described by Galay et al. [18] was performed. Briefly, a total of 35 larvae or nymphs were immersed in 40 μl of *HlGST*, *HlGST2*, or *HlGST 1/2* dsRNA dissolved to a concentration of 0.5 μg/μl in high purity water for 12 h. After 12 h, the dsRNA solution was removed, and the ticks were checked for mortality resulting from immersion. Ticks were also kept in vials sealed with cotton plug, placed in a glass chamber, and maintained at 25 °C and 80–85% relative humidity in an incubator for another 72 h. Total RNA was extracted from 5 ticks of each developmental stage and sex, and their cDNA was synthesized. The cDNA was subjected to RT-PCR with a Hot Start Pol system (Jena Bioscience, Jena, Germany) using *GST-*specific primers, such as HlGST RT forward and HlGST RT reverse primers, and HlGST2 RT forward and HlGST2 RT reverse primers (Table 1), following the manufacturer’s instructions. The PCR cycle profile was as follows: 94 °C for 8 min, 30 cycles of a denaturation step at 94 °C for 30 s, an annealing step at 68 °C for 60 s, and an extension step at 72 °C for 60 s. The PCR products were run in 1.5% TAE agarose gel and stained with ethidium bromide in TAE buffer. *Actin* was used as a loading control. The absence of bands corresponding to *HlGST* and *HlGST2* genes in their corresponding GST knockdown group demonstrates that silencing was successful (Additional file 1: Figure S1).

After confirmation of the knockdown, ticks were then exposed to sublethal doses of acaricides using the method described above. Mortality was checked after exposure. Ticks lying on their backs that could not turn over were considered dead.

### Statistical analysis

Welch’s t-test was used to analyze data from the enzymatic inhibition, real-time RT-PCR of ticks, and tick survival studies. A significant difference is defined as *P* < 0.05. All experiments were done at least twice.

## Results

### Interaction of recombinant GSTs with acaricides

Enzyme kinetic analysis in the presence or absence of acaricides was used to determine their ability to inhibit the activity of recombinant GST to catalyze the conjugation of CDNB to glutathione (GSH). The effect of acaricide interaction was determined by the change in the kinetic constants *V*_*max*_ and *K*_*m*_, in accordance with the procedure of Mathews and van Holde [19]. An inhibition that causes an increase in the *K*_*m*_ without a change in the *V*_*max*_ is a competitive type of inhibition. An inhibition in which the *K*_*m*_ is not affected by decreased V_max_ is a noncompetitive type of inhibition. An inhibition in which the *V*_*max*_ and *K*_*m*_ are decreased is an uncompetitive type of inhibition. In this experiment, flumethrin and cypermethrin showed uncompetitive inhibition of recombinant HlGST. On the other hand, chlorpyrifos and cypermethrin showed noncompetitive inhibition of recombinant HlGST2 (Table 2). Other acaricides, with the exception of coumaphos, did not show significant changes in the *V*_*max*_ and *K*_*m*_. Coumaphos significantly decreased the *K*_*m*_ of recombinant HlGST, indicating the apparent activation of the enzyme. These results demonstrated that the interactions of the recombinant GSTs with acaricides depend on the variety of acaricide.

**Table 2:** Enzyme kinetic constants of recombinant GSTs in the presence of different acaricides

Acaricide	Class	Recombinant HlGST			Recombinant HlGST2		
		*V*_*max*_	*K*_*m*_	Inhibition	*V*_*max*_	*K*_*m*_	Inhibition
None		11.70 ± 1.92	0.82 ± 0.14		14.72 ± 0.56	0.61 ± 0.20	
Flumethrin	Synthetic pyrethroids	4.26 ± 0.30*	0.48 ± 0.07*	UC	5.85 ± 0.73	1.70 ± 0.92	None
Cypermethrin	Synthetic pyrethroids	3.61 ± 1.65*	0.38 ± 0.04*	UC	2.20 ± 0.64*	0.36 ± 0.12	NC
Chlorpyrifos	Organophosphates	9.52 ± 4.21	0.55 ± 0.24	None	8.00 ± 1.08*	0.35 ± 0.25	NC
Ethion	Organophosphates	8.36 ± 0.87	0.58 ± 0.15	None	15.55 ± 1.02	0.37 ± 0.07	None
Coumaphos	Organophosphates	6.35 ± 2.40	0.48 ± 0.10*	None	9.68 ± 3.38	1.17 ± 0.31	None
Diazinon	Organophosphates	18.38 ± 5.43	1.51 ± 0.78	None	26.13 ± 5.33	1.59 ± 0.58	None
Amitraz	Formamidine	15.04 ± 1.66	0.87 ± 0.25	None	10.10 ± 2.55	0.51 ± 0.09	None
Ivermectin	Avermectin	10.94 ± 5.10	0.74 ± 0.36	None	14.64 ± 3.47	0.74 ± 0.31	None

*Abbreviations*: *UC* uncompetitive inhibition, *NC* noncompetitive inhibition

*P < 0.05 *vs* no acaricide

### Sublethal dose of flumethrin, chlorpyrifos and amitraz on different stages and strains of *H. longicornis*

Based on the results of enzyme kinetic analysis, flumethrin and chlorpyrifos were selected to analyze the importance of GSTs in their metabolism. Amitraz was also selected as a representative of the formamidine group. For adult parthenogenetic Okayama and bisexual Oita strains of ticks, the dilution of 40 μM proved to be the highest sublethal dose of flumethrin (Table 3), while 1 mM was the highest sublethal dose of chlorpyrifos (Table 4) and amitraz (Table 5). For nymphs, the dilution of 4 μM proved to be the highest sublethal dose of flumethrin (Table 3), while 100 μM was the highest sublethal dose of chlorpyrifos (Table 4) and amitraz (Table 5). For larvae, the dilution of 4 nM proved to be the highest sublethal dose of flumethrin (Table 3), while 100 nM was the highest sublethal dose of chlorpyrifos (Table 4) and amitraz (Table 5).

**Table 3:** Tick survival (in %) after exposure to different doses of flumethrin

	0	0.4 nM	4 nM	40 nM	400 nM	4 μM	40 μM	400 μM
Larva	90	100	90^a^	10	10	10	0	10
Nymph	100	nt	nt	nt	90	90^a^	50	10
P-adult	100	nt	nt	nt	100	100	100^a^	80
M-adult	100	nt	nt	nt	100	100	100^a^	80
F-adult	100	nt	nt	nt	100	100	100^a^	70

*Abbreviations*: *P-adult* parthenogenetic adult, *M-adult* adult male, *F-adult* adult female, *nt* not tested

^a^Maximum sublethal dose

The table is representative of three separate experiments showing approximately the same result

**Table 4:** Tick survival (in %) after exposure to different doses of chlorpyrifos

	0	1 nM	10 nM	100 nM	1 μM	10 μM	100 μM	1 mM	10 mM
Larva	100	100	90^a^	80	0	0	0	0	0
Nymph	100	nt	nt	nt	100	100	90^a^	0	0
P-adult	100	nt	nt	nt	nt	100	100	100^a^	0
M-adult	100	nt	nt	nt	nt	100	100	100^a^	0
F-adult	100	nt	nt	nt	nt	100	100	100^a^	0

*Abbreviations*: *P-adult* parthenogenetic adult, *M-adult* adult male, *F-adult* adult female, *nt* not tested

^a^Maximum sublethal dose

The table is representative of three separate experiments showing approximately the same result

**Table 5:** Tick survival (in %) after exposure to different doses of amitraz

	0	1 nM	10 nM	100 nM	1 μM	10 μM	100 μM	1 mM	10 mM
Larva	100	90	100^a^	80	0	0	0	0	0
Nymph	100	nt	100	100	100	90^a^	80	50	0
P-adult	100	nt	nt	nt	nt	100	100	90^a^	0
M-adult	100	nt	nt	nt	nt	100	100	100^a^	0
F-adult	100	nt	nt	nt	nt	100	100	90^a^	0

*Abbreviations*: *P-adult* parthenogenetic adult, *M-adult* adult male, *F-adult* adult female, *nt* not tested

^a^Maximum sublethal dose

The table is representative of three separate experiments showing approximately the same result

### Effect of flumethrin, chlorpyrifos and amitraz on the gene and protein expression of GSTs of parthenogenetic ticks

**Fig. 1:**
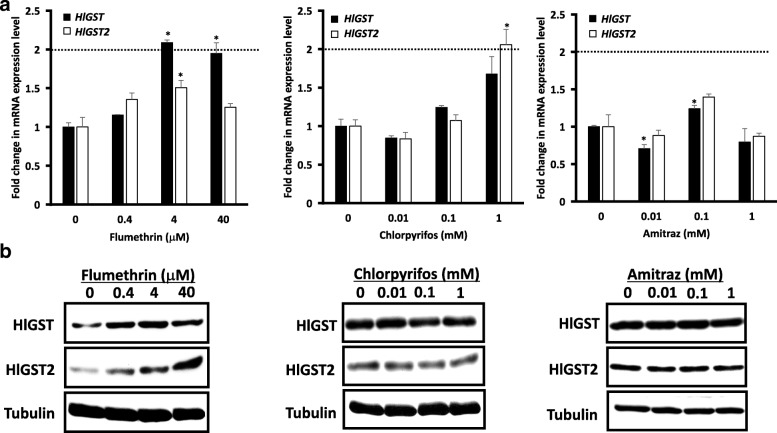
Gene (**a**) and protein (**b**) expression of GSTs of adult parthenogenetic ticks exposed to sublethal doses of flumethrin, chlorpyrifos and amitraz. **a** Total mRNA was extracted from adult ticks and transcribed to cDNA before real-time RT-PCR. *P0* primers were used as controls. The error bar represents the mean ± standard deviation. *P < 0.05: significantly different by Welch’s t*-*test as compared to no treatment. The dotted line indicates overexpression. Overexpression is determined if there is at least a 2-fold increase in expression level, as shown by Bhattacharjee et al. [42]. **b** Proteins were prepared from adult ticks exposed to sublethal doses of flumethrin, chlorpyrifos and amitraz. Antiserum against tubulin was used as a control for Western blot analysis. Western blot analysis results are shown as representative data of three separate experiments showing the same trend

The effect of flumethrin, chlorpyrifos and amitraz on the mRNA expression level of parthenogenetic female ticks were also tested (Fig. 1a). Exposure to 4 μM and 40 μM flumethrin resulted in the overexpression of *HlGST. HlGST2* genes at 0.4 μM and 40 μM flumethrin exposure did not show any significant increase in mRNA expression, but they showed a significant increase at 4 μM flumethrin exposure (*t*_(2)_ = 4.67, *P* = 0.043). Chlorpyrifos exposure did not result in any significant change in *HlGST* gene expression. On the other hand, the *HlGST2* gene was overexpressed when ticks were exposed to 1 mM chlorpyrifos. Although amitraz exposure did not cause overexpression in either *GST*, *HlGST* showed a significant decrease in expression at a concentration of 0.01 mM (*t*_(2)_ = 7.43, *P* = 0.018), while it increased significantly at a 0.1 mM concentration (*t*_(2)_ = 6.92, *P* = 0.020). *HlGST2* genes did not show any significant change when ticks were exposed to amitraz. These results demonstrated that flumethrin and chlorpyrifos trigger the overexpression of *HlGST* and *HlGST2* genes, respectively*.* This might then indicate their possible role in the metabolism of these acaricides.

GST proteins of adult parthenogenetic *H. longicornis* ticks exposed to sublethal doses of flumethrin, chlorpyrifos, and amitraz were detected by Western blot analysis (Fig. 1b). The expression of HlGST protein was induced with exposure to 0.4 μM, 4 μM and 40 μM flumethrin. The protein expression of HlGST2 increased in a dose-dependent manner. Chlorpyrifos and amitraz at sublethal doses (0.01, 0.1 and 1 mM) did not cause any significant change in the expression of GST proteins. These results demonstrate that acaricides utilize GST proteins differently.

To be able to determine whether larval GSTs are induced in the same manner as the parthenogenetic female, parthenogenetic larvae exposed to sublethal doses of flumethrin (0, 0.4 and 4 nM) and chlorpyrifos (0, 10 and 100 nM) were checked for their gene and protein expression. Similar upregulation of *HlGST* genes and proteins was observed upon larval exposure to sublethal doses of flumethrin (Additional file 2: Figure S2). Unlike in adults, both the *HlGST2* gene and protein were upregulated upon larval exposure to a sublethal dose of chlorpyrifos (Additional file 2: Figure S2). This indicates that the utilization of GSTs could vary between tick stages.

### Effect of *GST* knockdown on different stages of parthenogenetic ticks upon exposure to flumethrin and chlorpyrifos

**Fig. 2:**
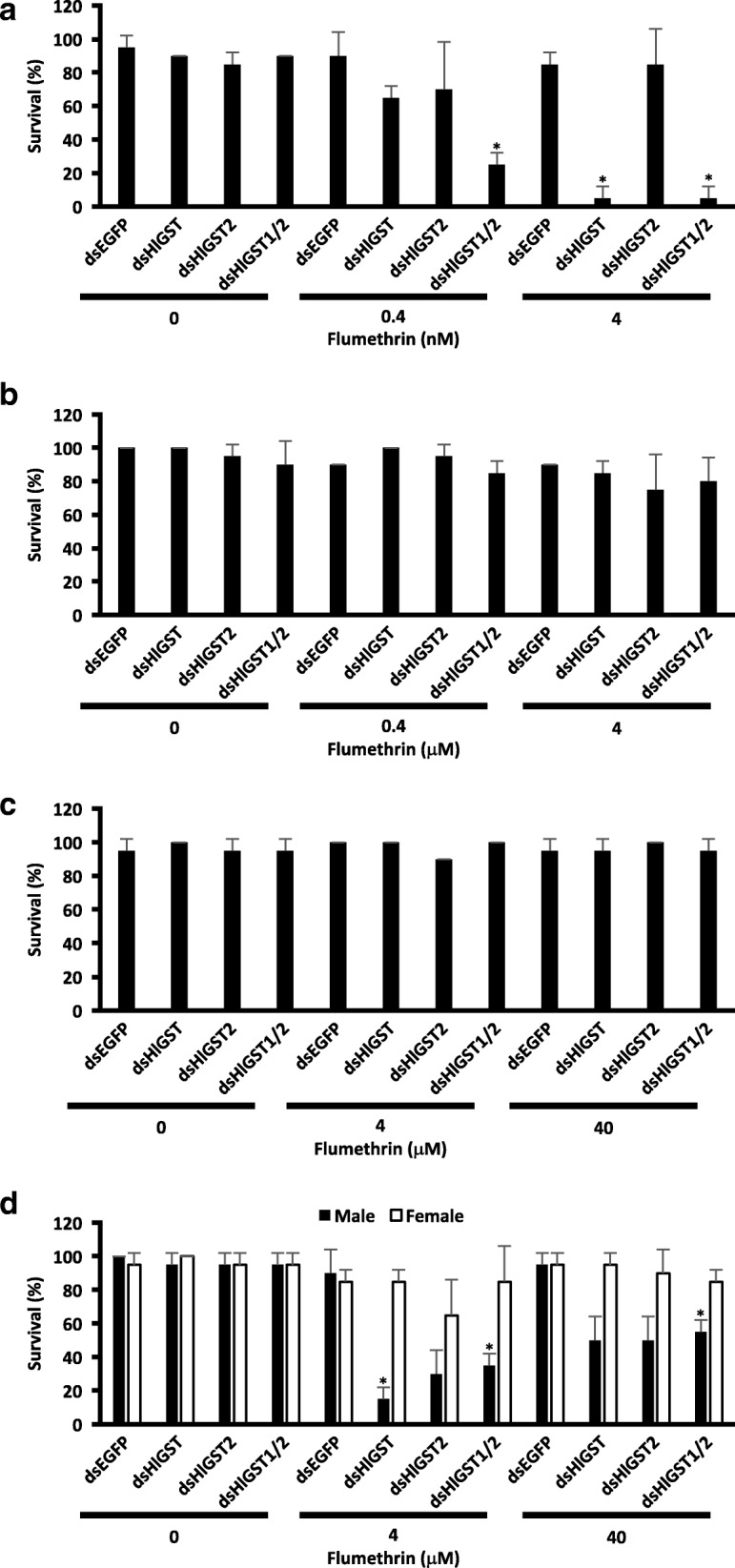
Tick survival upon exposure to sublethal doses of flumethrin. Parthenogenetic larva (**a**), nymph (**b**), adult (**c**) and bisexual adult (**d**) ticks were exposed to sublethal doses of flumethrin for 48 h. Ticks lying on their backs that could not turn over were considered dead. *P < 0.05: significantly different by Welch’s t*-*test as compared to the *EGFP* knockdown of the same acaricide concentration

**Fig. 3:**
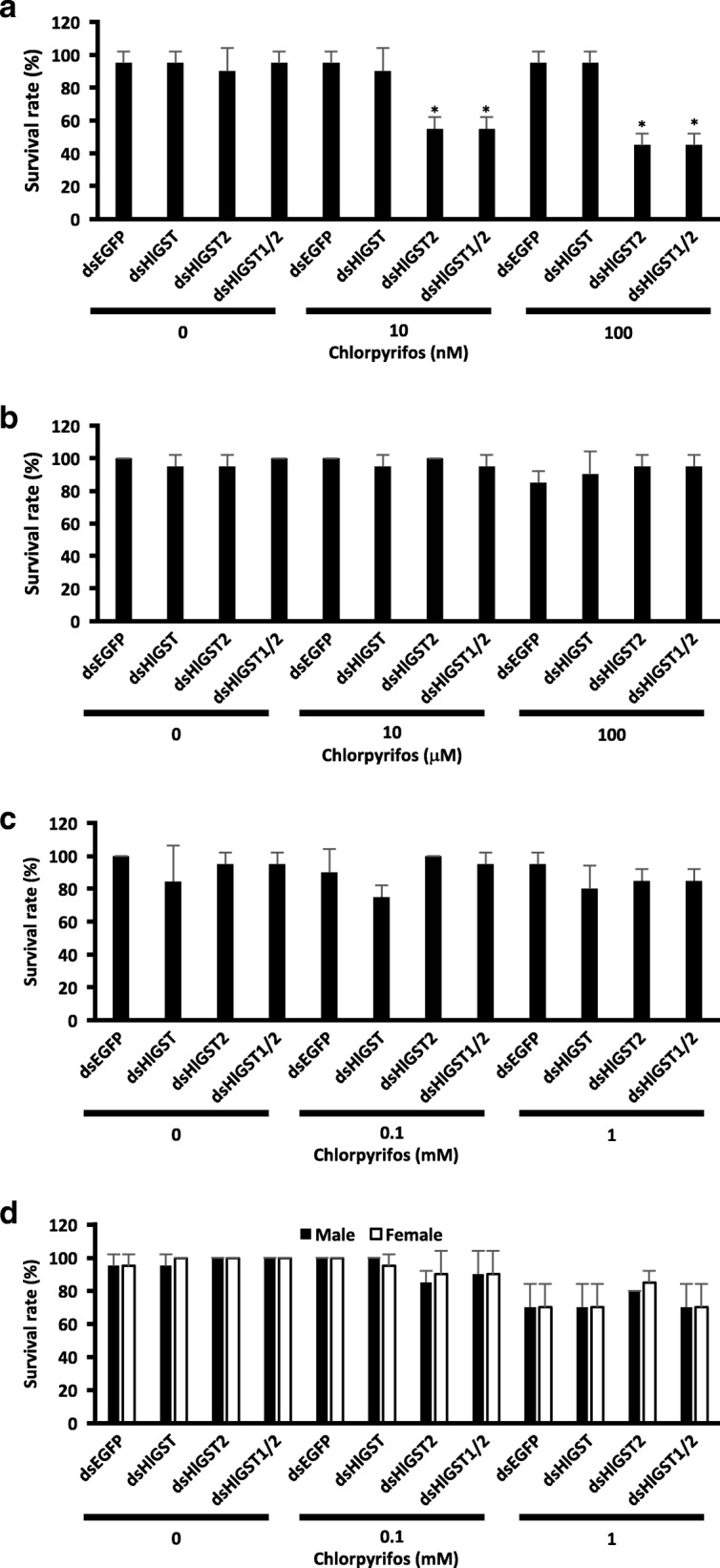
Tick survival upon exposure to sublethal doses of chlorpyrifos. Parthenogenetic larva (**a**), nymph (**b**), adult (**c**) and bisexual adult (**d**) ticks were exposed to sublethal doses of chlorpyrifos for 48 h. Ticks lying on their backs that could not turn over were considered dead. *P < 0.05: significantly different by Welch’s t-test as compared to the *EGFP* knockdown of the same acaricide concentration

To further establish the importance of GST in acaricide detoxification, *GST* knockdown experiments were performed, and *GST* knockdown ticks were then exposed to different sublethal doses of flumethrin and chlorpyrifos. Nymphs and adults showed no significant increase in mortality in *GST* knockdowns as compared to *EGFP* knockdown groups (Figs. 2 and 3). The knockdown of *HlGST* in larvae resulted in the death of almost all larvae tested in 4 nM flumethrin (Fig. 2a). Additionally, a significant decrease in survival was also observed when both *HlGST* and *HlGST2* were knocked down and larvae were exposed to 0.4 nM flumethrin as compared to *HlGST* knockdown alone (*t*_(2)_ = 5.66, *P* = 0.030). On the other hand, the knockdown of *HlGST2* and both *HlGST* and *HlGST2* caused a significant increase in the mortality of larvae exposed to 10 μM (*HlGST2*: *t*_(2)_ = 5.66, *P* = 0.030; *HlGST* and *HlGST2*: *t*_(2)_ = 5.66, *P* = 0.030) and 100 μM chlorpyrifos (*HlGST2*: *t*_(2)_ = 7.07, *P* = 0.019; *HlGST* and *HlGST2*: *t*_(2)_ = 7.07, *P* = 0.019) (Fig. 3a). These results showed that HlGST is vital for the survival of larval ticks against sublethal doses of flumethrin, while HlGST2 is important in larval tick survival against chlorpyrifos.

### Effect of *GST* knockdown on different sexes of ticks upon exposure to flumethrin and chlorpyrifos

**Fig. 4:**
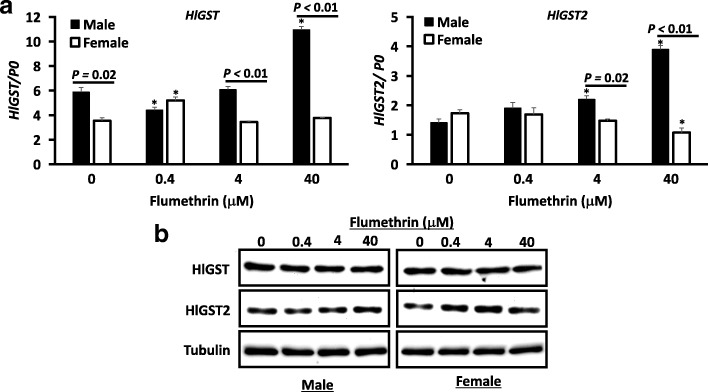
Gene (**a**) and protein (**b**) expressions of HlGST of male and female ticks upon exposure to sublethal doses of flumethrin. Ticks were exposed to sublethal doses (0, 0.4, 4 and 40 μM) of flumethrin for 48 h. **a** Total mRNA extracted from ticks was transcribed to cDNA for real-time RT-PCR. *P0* primers were used as controls. The error bar represents the mean ± standard deviation. *P < 0.05: significantly different by Welch’s t-test as compared to no treatment. **b** Proteins were prepared from ticks exposed to sublethal doses of acaricides. Antiserum against tubulin was used as a control for Western blot analysis. The Western blot analysis results are shown as representative data of three separate experiments showing the same trend

In mammals, as well as insects, sexual differences in GST expression have been observed [20–22]; therefore, gene knockdown experiments with subsequent exposure to acaricides were also performed with male and female *H. longicornis* ticks to determine whether sex is a factor in tick survival against flumethrin and chlorpyrifos. Results have shown that the knockdown of *HlGST* and *HlGST2* with subsequent exposure to sublethal doses of flumethrin leads to a significant increase in the mortality of male ticks (4 μM: *t*_(2)_ = 4.92, *P* = 0.039; 40 μM: *t*_(2)_ = 5.66, *P* = 0.030) (Fig. 2d). To check whether males and females have the same induction response, real-time RT-PCR and Western blot analysis were performed to check the expression levels of HlGST and HlGST2. Interestingly, although both *GST* genes are induced upon exposure to flumethrin, protein expressions were constant in male ticks (Fig. 4). On the other hand, female ticks showed no induction in either gene or protein expression. These results showed that HlGST is vital for the survival of male ticks against sublethal doses of flumethrin. These results also demonstrated that different strains of ticks have different induction responses to acaricides.

## Discussion

The predominant tick-control measure is the use of acaricides. Ticks make use of several mechanisms to metabolize these compounds. Therefore, several factors could be considered in the development of new tick-control strategies, such as the type of acaricide and its schedule of application. It is then important to understand the mechanism through which ticks metabolize these substances. Interference with these mechanisms would make the tick more prone to an acaricide and, eventually, lead to a more efficient tick control method [6, 23].

In this study, the role of two kinds of GSTs in the detoxification of several acaricides was investigated. We previously identified and expressed two mu-class GSTs from the hard tick *H. longicornis* [9], on which we used expressed recombinant GSTs to perform enzyme kinetic analysis in the presence of acaricides. The inhibition of GST activity by acaricides or insecticides has been observed previously [3, 24]. Differences in the type of inhibitions demonstrated that each GST isoenzyme has a specific profile of interaction with chemical compounds, even though the same chemical compounds would have different interactions with GST isoenzymes [3].

In cases of uncompetitive inhibition, inhibitors such as flumethrin and cypermethrin (Table 2) would only bind to an enzyme-substrate complex. This binding could also result in an irreversible interaction that may inactivate the enzyme [19]. This inhibition also could be a result of the combination of GST and acaricide rather than conjugation of the acaricide with a reduced glutathione. This kind of binding of GSTs with pyrethroids was also observed in insects such as *Tenebrio* sp. and *Aedes* sp. Pyrethroids are believed to bind to the enzyme’s active site but did not yield a conjugated product. This suggests that the enzyme acts as a binding protein rather than a conjugating protein [25, 26]. This type of combination reaction was also observed in other detoxification enzymes, such as esterase. *Tetranychus cinnabarinus* esterase (TCE2) protein combined with abamectin rather than hydrolyzing it. It is believed that this binding decreased or delayed the noxious compound from reaching its target site; therefore, it is still considered an important mechanism in the metabolism of abamectin [27].

Noncompetitive inhibition occurs when an inhibitor, such as cypermethrin or chlorpyrifos (Table 2), binds to a non-substrate binding site. The presence of a non-substrate binding site was also observed in the pi class of GSTs [8]. In this class of GSTs, the presence of low-affinity and high-affinity binding sites of the enzyme for bilirubin has been observed. Also, the inhibition of GST activity by bromosulfophthalein was attributed to the non-substrate binding site [8]. Moreover, the noncompetitive inhibition of flumethrin was also observed in the recombinant *Rhipicephalus* (*Boophilus*) *microplus* GST [3].

In the present study, recombinant HlGST was activated by coumaphos through the lowering of the *K*_*m*_ (Table 2). This kind of activation has also been observed in the recombinant *R. microplus* GST, wherein its activity was activated by coumaphos, the biological significance of which is still uncertain and could be the subject of a future study [3]. Since the *in vitro* reaction showed a potential role of GSTs in interacting with acaricides, several types of acaricides that have interacted with GSTs were testedor their effect on the GST gene and protein expression levels of ticks exposed to acaricides.

Dose-dependent gene expression that led to overexpression was observed on the *HlGST* gene when adult ticks were exposed to sublethal doses of flumethrin (Fig. 1a). Although pyrethroids are not supposed to be substrates of GSTs, our enzyme kinetic analysis has shown the ability of recombinant HlGST to bind with flumethrin. This binding or sequestering mechanism of GSTs could give passive protection by either decreasing the level of free pyrethroids or facilitating the binding of other enzymes with it [26, 28]. This binding could be facilitated by alkyl or aryl hydrogen groups in the pyrethroids that could interact with GSTs. Also, the role of GST in detoxifying lipid peroxidation products caused by pyrethroids should be considered in determining the role of GST in the metabolism of an acaricide [29]. The overexpression of *GST* genes of *H. longicornis* upon exposure to flumethrin was also observed by Hatta et al. [16]. The overexpression of mu-class *GST* genes was also observed in mites after exposure to pyrethroids [28, 30]. Although the larva has a higher basal GST expression as compared with the adult [9], the ability of flumethrin to induce *HlGST* gene and protein expression remains the same. Interestingly, when the bisexual strain of *H. longicornis* was used, a different expression pattern was observed (Fig. 4a). No induction of *GST* genes was observed in the female bisexual strain as compared with the parthenogenetic *H. longicornis.* In the case of bisexual males, the induction of gene expression was observed for both *HlGST* and *HlGST2* genes. This different expression of GSTs among arthropod strains was also observed in *Anopheles gambiae*, in which two strains (G3 and ZANDS) demonstrated different expressions in response to DDT. It is believed that GST expression is greatly influenced by environmental factors. Environmental factors, especially those that could confer resistance to a pesticide, include temperature, the type and dose of pesticide to which the arthropod has been exposed, and even the solvent quality used to dilute the pesticide [31]. The continuous effects of these environmental factors have already resulted in changes at the genetic level [11, 32]. In the case of sex, cDNA analysis showed no difference between parthenogenetic, male and female ticks (Additional file 3: Figure S3). It should be noted that male and female ticks have demonstrated different gene expression patterns upon exposure to sublethal doses of flumethrin (Fig. 4a). Although the exact mechanism of the difference in male and female GST gene induction still remains unknown, differences in GST activity have been observed previously in mosquitoes [20]. In the locust, different *GST* gene expressions were also observed between spermaries and ovaries [21]. Male and female ticks could have different *GST* gene composition levels. In this way, when ticks are exposed to flumethrin, a *GST* with a higher activity against flumethrin would result in increased transcription. Therefore, if there is another unknown GST that has higher activity than the identified *GST*s, the unknown GST could have proliferated instead of the identified ones. Therefore, if there is an unknown GST gene present in the female and absent in the male, it would result in different transcriptions in the known *GST* genes. On the other hand, when mosquitoes were exposed to pyrethroids, the overexpression of multiple *cytochrome P450* genes was observed aside from the upregulated *GST* genes [33, 34].

The *HlGST2* gene showed overexpression at the highest sublethal dose of chlorpyrifos (Fig. 1a). However, it is well known that organophosphates are metabolized by cytochrome P450 monooxygenases and hydrolases. Specifically, chlorpyrifos is activated to chlorpyrifos oxon by cytochrome P450 enzymes before being deethylated or dearylated. The metabolism of chlorpyrifos could yield metabolites that could be subjected to GSH conjugation by GSTs [10]. Therefore, GSTs could have an indirect role in organophosphate detoxification. In *R.* (*B.*) *microplus*, the overexpression of *GST* was observed when ticks were exposed to coumaphos [35]. On the other hand, chlorpyrifos has shown the ability to overexpress the *GST* gene of the migratory locust, cotton leaf worm and rice plant hopper [36–38]. Interestingly, *HlGST2* genes appear to be maintained at lower concentrations of chlorpyrifos (Fig. 1a). The noninduction of *HlGST2* at low doses of chlorpyrifos could be because of the organism’s ability to specifically produce the appropriate *GST* genes depending on its needs. Different responses of GSTs to chlorpyrifos were also observed in migratory locusts [36]. On the other hand, amitraz did not show any effect on the *GST* gene expression level. Since amitraz is considered to be an agonist in the octopaminergic system of arthropods [7], this mimicry could have resulted in its non-recognition as a foreign or xenobiotic compound and, therefore, not a metabolite of the GSTs.

In accordance with the *GST* gene expression level, the protein expression level of HlGST2 also increased depending on the concentration of flumethrin (Fig. 1b). This also held true in larvae of ticks exposed to sublethal doses of chlorpyrifos. When ticks were exposed to a sublethal dose of flumethrin, HlGST protein initially increased but appeared to maintain its expression when the dosage of flumethrin was increased, even though gene expression increased dramatically (Fig. 1b). On the other hand, no difference was observed in the protein expression level regardless of the concentration when adult ticks were exposed to sublethal doses of chlorpyrifos (Fig. 1b). It has always been believed that GST proteins are transcriptionally regulated [39]. Based on our results and previous studies, GST not only functions as a conjugate with GSH but also as a binding protein, wherein they “sacrifice” themselves by binding covalently with reactive compounds [40]. The fate of the GST conjugates is yet to be demonstrated. They could possibly be released from the cytoplasm, as some studies have shown the ability of GST to be secreted or to move across the plasma membrane through the facilitation of MRP [22, 41]. This could be why non-substrates that bind with GST, such as flumethrin with HlGST or chlorpyrifos with HlGST2, did not have a drastic increase in protein expression, as GST proteins are being released from the cell after being bound to flumethrin or chlorpyrifos.

To further understand the role of GSTs in flumethrin and chlorpyrifos metabolism, RNAi was performed on GST genes, and ticks at different stages were exposed to sublethal doses of the acaricides. No significant differences in mortality were observed in the knockdown groups of adult female and nymph ticks exposed to sublethal doses of flumethrin and chlorpyrifos (Figs. 2 and 3). Other GST isoenzymes could have possibly compensated for the silenced *GST* [42]. Several tick species have demonstrated the presence of multiple isoenzymes of GSTs. Thirty-five genes of GSTs, of which 14 belong to the mu-class GST were shown in an *in silico* analysis of the *Ixodes scapularis* gene database [43]. Multiple GSTs have also been found in *Dermacentor variabilis* and *Rhipicephalus* (*Boophilus*) *annulatus* [3, 44]. Interestingly, the *HlGST* knockdown in male ticks resulted in increased susceptibility to flumethrin (Fig. 2d). This could mean that HlGST could be the main GST in the male tick’s detoxification mechanism against flumethrin. However, further testing and studies need to be conducted on the different detoxification mechanisms between male and female ticks. Notably, the larval stage of ticks also showed increased susceptibility to the effects of sublethal doses of flumethrin and chlorpyrifos. The importance of the GST system at the early stage of development was also observed in insects such as *Tenebrio* and *Anopheles,* as well as the red mite *Panonychus*, wherein increased expression and/or activity was observed at the younger stage of development as compared with the adult [20, 28, 45, 46]. Our previous results have also shown relatively higher GST protein expression in the unfed larval stages of ticks as compared to the unfed nymph and adult *H. longicornis* [9]*.* Since the younger stages do not have a well-developed system of protection, such as integuments, it is possible that the GST system is vital for protecting larvae by detoxification.

Flumethrin may not be readily conjugated to glutathione by GSTs; however, GST could bind to flumethrin to decrease its ability to reach the tick nerve cell membrane. This, in turn, could result in a reduced toxic effect of flumethrin. The knockdown of *HlGST* in larvae and adult male ticks also could have resulted in increased intracellular flumethrin, eventually leading to cellular toxemia. The knockdown of GST genes in *Rhipicephalus sanguineus* also leads to increased mortality upon exposure to a sublethal dose of pyrethroids [6]. In mites, exposure to pyrethroids after GST inhibition also resulted in increased susceptibility to acaricides [30]. Higher mortality was also observed when both *GST*s were knocked down, as compared to the knockdown of *HlGST* alone, when larvae were exposed to a sublethal dose of flumethrin. This could possibly show the ability of HlGST2 to compensate, to a certain degree, for the loss of HlGST. On the other hand, the metabolism of chlorpyrifos leads to the production of toxic metabolites [10]. These metabolites could have increased when *HlGST2* is knocked down in larvae. The abundance of these metabolites could have resulted in intracellular toxicity and, eventually, the death of ticks. The same increase in susceptibility to chlorpyrifos upon *GST* knockdown has also been observed in migratory locusts [36].

## Conclusions

In conclusion, GSTs have been known to be involved in the detoxification of xenobiotic compounds [24]. HlGST could play an important role in the detoxification of pyrethroids such as flumethrin, wherein the inhibition of enzymatic activity was observed. *HlGST* gene expression was also induced by sublethal doses of flumethrin, and the knockdown of *HlGST* resulted in the increased susceptibility of larvae and male ticks to flumethrin. On the other hand, HlGST2 plays an important function in the metabolism of organophosphates, such as chlorpyrifos. Chlorpyrifos inhibited the GST activity of recombinant HlGST2. *HlGST2* gene induction upon exposure to sublethal doses of chlorpyrifos was also observed. More importantly, larval susceptibility to chlorpyrifos significantly increased upon *HlGST2* knockdown. The data also showed that GSTs have a more important role in larval survival as compared to ticks at other stages upon exposure to sublethal doses of acaricides. We have also demonstrated that environmental factors as well as GST pool composition could play a role in the ability of acaricides to induce GST gene expression as observed in male and female ticks. These results have shown the importance of specific GSTs in the acaricide detoxification mechanism and could be considered by tick scientists in the development of new tick control strategies.

## Additional files

Additional file 1: **Figure S1.** RT-PCR of knockdown ticks. Total RNA was extracted from whole *GST* and *EGFP* knockdown ticks 4 days post-injection/immersion to dsRNA. cDNA was synthesized and subjected to RT-PCR. PCR products were run on 1.5% TAE agarose gel and stained with ethidium bromide. *Actin* was used as a loading control. (PDF 1275 kb)

Additional file 2: **Figure S2.** Gene (**a**) and protein (**b**) expressions of larval ticks upon exposure to sublethal doses of flumethrin and chlorpyrifos. Ticks were exposed to sublethal doses of flumethrin and chlorpyrifos for 48 h. **a** Total mRNA was extracted from ticks, and cDNA was then transcribed for real-time RT-PCR. *P0* primers were used as a control. The error bar represents the mean ± standard deviation. *P < 0.05: significantly different by Welch’s t*-*test as compared to no treatment. The dotted line indicates overexpression. Overexpression is determined if there is at least a twofold increase in expression level, as shown by Bhattacharjee et al. [42]. **b** Proteins were prepared from ticks exposed to sublethal doses of acaricides. Antiserum against tubulin was used as a control for Western blot analysis. Western blot analysis results are shown as representative data of three separate experiments showing the same trend. (PDF 817 kb)

Additional file 3: **Figure S3.** Multiple sequence alignments of the nucleotide sequence of *HlGST* (**a**) and *HlGST2* (**b**) of parthenogenetic, male and female ticks. Sequencing was performed using an automated sequencer (ABI PRISM 3100, Genetic Analyzer; Applied Biosystems, Foster City, CA, USA). Multiple sequence alignments of *GST* genes were done using MAFFT version 7 program (www.mafft.cbrc.jp). Asterisks indicate identity. Start and stop codons are in red. (ZIP 69 kb)

## Abbreviations

CDNB: 1-chloro-2,4-dinitrobenzene
EGFP: Enhanced green fluorescent protein
GST: Glutathione S-transferase
HlGST: GST of *H. longicornis*
MRP: Multidrug-resistance-related protein
PBS: Phosphate-buffered saline
PVDF: Polyvinylidene difluoride
RNAi: RNA interference
SDS-PAGE: Sodium dodecyl sulphate polyacrylamide gel electrophoresis
SFTSV: Severe fever with thrombocytopenia virus
TAE: Tris-acetate-EDTA
TCE2: *T. cinnabarinus* esterase
