# Supplementary figures and images for: Glutathione S-transferases play a role in the detoxification of flumethrin and chlorpyrifos in Haemaphysalis longicornis
Source: Parasit Vectors. 2018 Aug 9;11:460. doi: 10.1186/s13071-018-3044-9 (PMC6085608; doi:10.1186/s13071-018-3044-9)

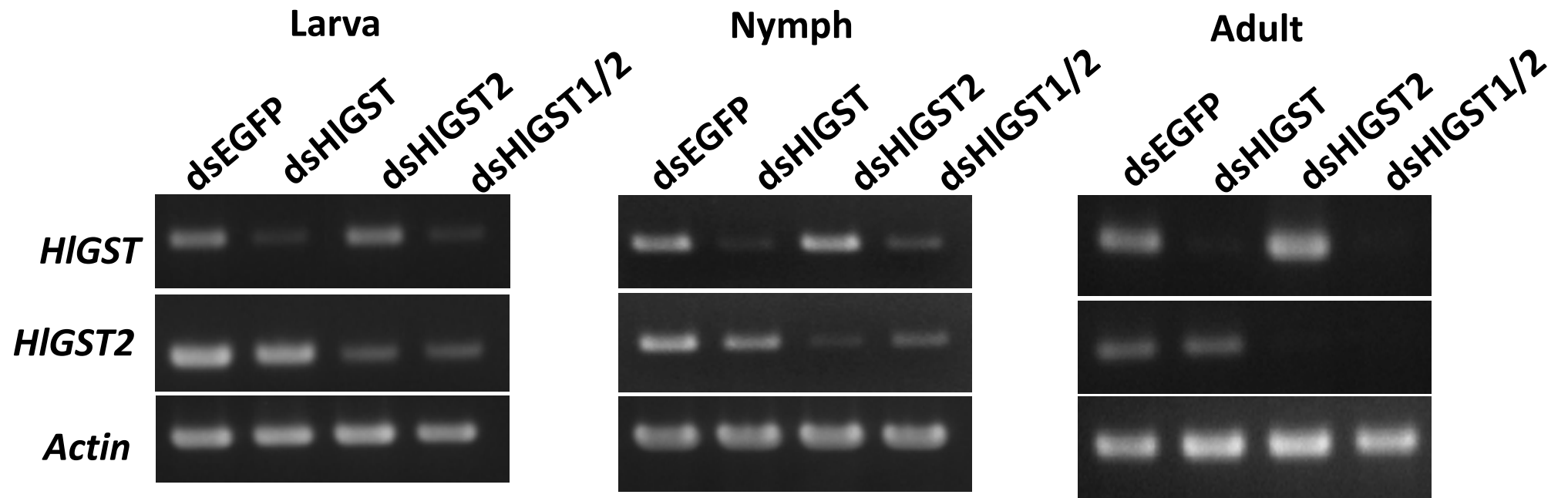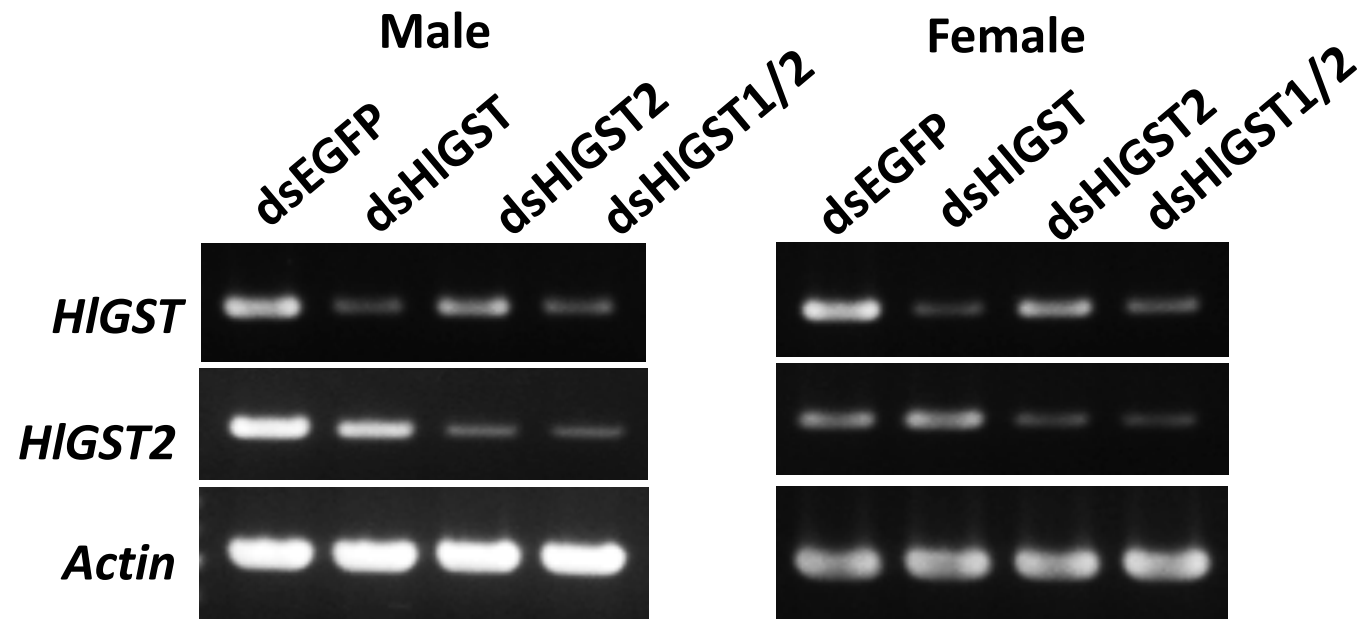

Supplement: Supplementary file 1 — Figure S1. RT-PCR of knockdown ticks. Total RNA was extracted from whole GST and EGFP knockdown ticks 4 days post-injection/immersion to dsRNA. cDNA was synthesized and subjected to RT-PCR. PCR products were run on 1.5% TAE agarose gel and stained with ethidium bromide. Actin was used as a loading control. (PDF 1275 kb) [file 13071_2018_3044_MOESM1_ESM.pdf]

**A**

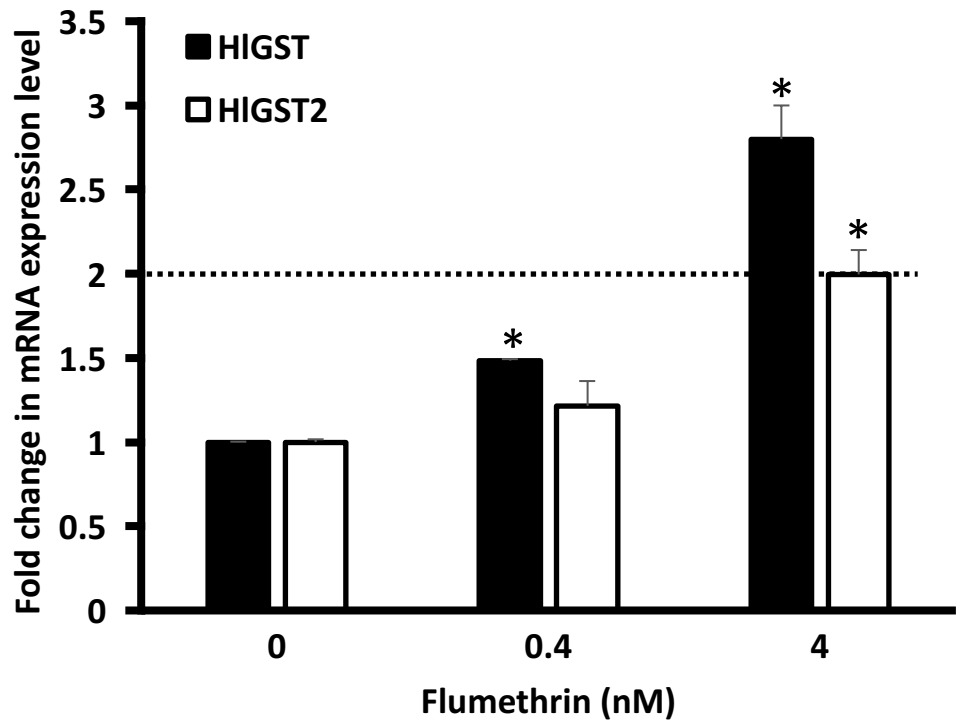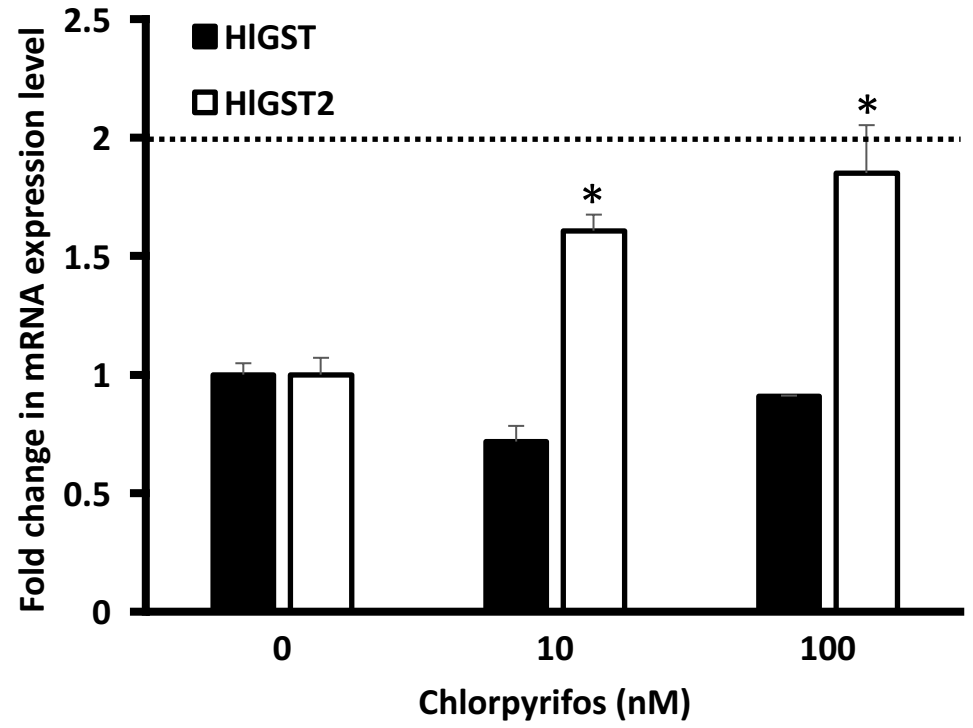

**B**

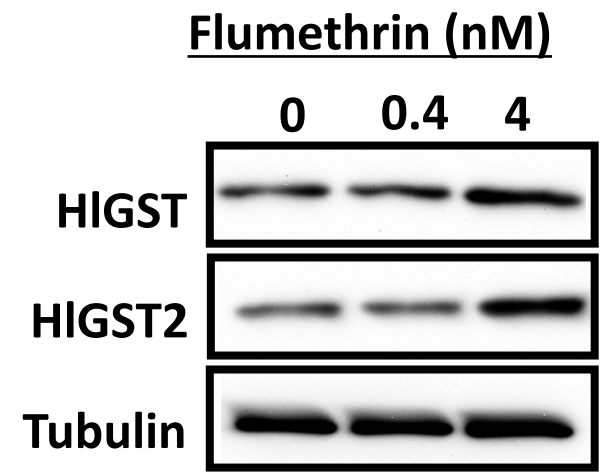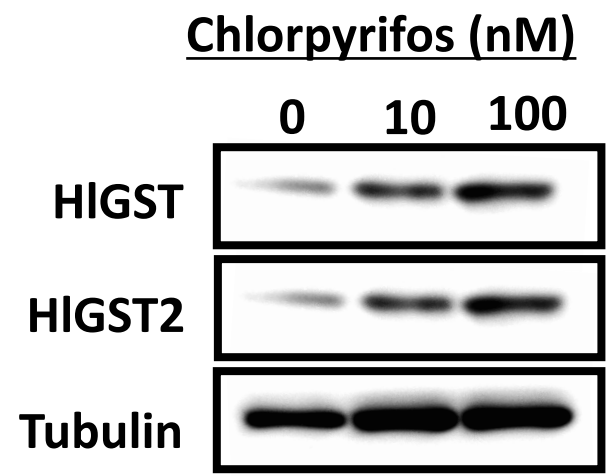

Supplement: Supplementary file 2 — Figure S2. Gene (a) and protein (b) expressions of larval ticks upon exposure to sublethal doses of flumethrin and chlorpyrifos. Ticks were exposed to sublethal doses of flumethrin and chlorpyrifos for 48 h. a Total mRNA was extracted from ticks, and cDNA was then transcribed for real-time RT-PCR. P0 primers were used as a control. The error bar represents the mean ± standard deviation. *P < 0.05: significantly different by Welch’s t-test as compared to no treatment. The dotted line indicates overexpression. Overexpression is determined if there is at least a twofold increase in expression level, as shown by Bhattacharjee et al. [42]. b Proteins were prepared from ticks exposed to sublethal doses of acaricides. Antiserum against tubulin was used as a control for Western blot analysis. Western blot analysis results are shown as representative data of three separate experiments showing the same trend. (PDF 817 kb) [file 13071_2018_3044_MOESM2_ESM.pdf]
