# Supplementary material for: Glutathione S-transferases play a role in the detoxification of flumethrin and chlorpyrifos in Haemaphysalis longicornis
Source: Parasit Vectors. 2018 Aug 9;11:460. doi: 10.1186/s13071-018-3044-9 (PMC6085608; doi:10.1186/s13071-018-3044-9)
Supplement: Supplementary file 3 — Figure S3. Multiple sequence alignments of the nucleotide sequence of HlGST (a) and HlGST2 (b) of parthenogenetic, male and female ticks. Sequencing was performed using an automated sequencer (ABI PRISM 3100, Genetic Analyzer; Applied Biosystems, Foster City, CA, USA). Multiple sequence alignments of GST genes were done using MAFFT version 7 program (www.mafft.cbrc.jp). Asterisks indicate identity. Start and stop codons are in red. (ZIP 69 kb) [file 13071_2018_3044_MOESM3_ESM.zip › Additional File 3 Figure S3a 20180726.pdf]

a

```
Parthenogenetic gattcgcttcttggatcttggcgggtgttgtgaacagtcgctgcattttcaactgcttta
Male            ------cttcttggatcttggcgggtgttgtgaacagtcgctgcattttcaactgcttta
Female          --------------------------------aacagtcgctgcattttcaactgcttta
                                                ****************************

Parthenogenetic accatggctcctattctcggctactgggacattcgtggactggcacagccaatccgcctg
Male            accatggctcctattctcggctactgggacattcgtggactggcacagccaatccgcctg
Female          accatggctcctattctcggctactgggacattcgtggactggcacagccaatccgcctg
                ************************************************************

Parthenogenetic ctgcttgcccacgctgacgtcaaggtggaggacaagcgctactcatgcggacccccctccg
Male            ctgcttgcccacgctgacgtcaaggtggaggacaagcgctactcatgcggacccccctccg
Female          ctgcttgcccacgctgacgtcaaggtggaggacaagcgctactcatgcggacccccctccg
                ************************************************************

Parthenogenetic gattttgaccgcagcgcctggctcaaggagaaacacaccctgggcctggagttccccaac
Male            gattttgaccgcagcgcctggctcaaggagaaacacaccctgggcctggagttccccaac
Female          gattttgaccgcagcgcctggctcaaggagaaacacaccctgggcctggagttccccaac
                ************************************************************

Parthenogenetic ctgccttactacattgatggggacgtgaagctcacccagagcatggctattctgcgctac
Male            ctgccttactacattgatggggacgtgaagctcacccagagcatggctattctgcgctac
Female          ctgccttactacattgatggggacgtgaagctcacccagagcatggctattctgcgctac
                ************************************************************

Parthenogenetic cttgcccgcaagcatggactggatggcaagacagaggccgaaaagcaacgggtcgacgtc
Male            cttgcccgcaagcatggactggatggcaagacagaggccgaaaagcaacgggtcgacgtc
Female          cttgcccgcaagcatggactggatggcaagacagaggccgaaaagcaacgggtcgacgtc
                ************************************************************

Parthenogenetic acggagcagcagtttgccgacttccgcatgaactgggttcgcatgtgctacaacccagac
Male            acggagcagcagtttgccgacttccgcatgaactgggttcgcatgtgctacaacccagac
Female          acggagcagcagtttgccgacttccgcatgaactgggttcgcatgtgctacaacccagac
                ************************************************************

Parthenogenetic tttgacaagctcaaggtcgactacctcaagaacttgccagatgcgctgaagagcttctca
Male            tttgacaagctcaaggtcgactacctcaagaacttgccagatgcgctgaagagcttctca
Female          tttgacaagctcaaggtcgactacctcaagaacttgccagatgcgctgaagagcttctca
                ************************************************************

Parthenogenetic gagtaccttgggaagcacaagttcttcgctggcgaccatgtcacctacgttgacttcatc
Male            gagtaccttgggaagcacaagttcttcgctggcgaccatgtcacctacgttgacttcatc
Female          gagtaccttgggaagcacaagttcttcgctggcgaccatgtcacctacgttgacttcatc
                ************************************************************

Parthenogenetic gcttacgagatgctggctcagcacctcctctttgctccggactgcctgaaggatttcccc
Male            gcttacgagatgctggctcagcacctcctctttgctccggactgcctgaaggatttcccc
Female          gcttacgagatgctggctcagcacctcctctttgctccggactgcctgaaggatttcccc
                ************************************************************

Parthenogenetic aacctgaaggcctttgtggaccgcgttgaggctctcccccacgtggcggcctacctgaag
Male            aacctgaaggcctttgtggaccgcgttgaggctctcccccacgtggcggcctacctgaag
Female          aacctgaaggcctttgtggaccgcgttgaggctctcccccacgtggcggcctacctgaag
                ************************************************************

Parthenogenetic tctgacaagtgcatcagctggcccctcaacggcgacatggctagcttcggcagcaggctg
Male            tctgacaagtgcatcagctggcccctcaacggcgacatggctagcttcggcagcaggctg
Female          tctgacaagtgcatcagctggcccctcaacggcgacatggctagcttcggcagcaggctg
                ************************************************************

Parthenogenetic cagaagaagccgtgaacagcacttcatacccactgtccgctttggcgtttgcgttccccc
Male            cagaagaagccgtgaacagcacttcatacccactgtccgctttggcgtttgcgttccccc
Female          cagaagaagccgtgaacagcacttcatacccactgtccgctttggcgtttgcgttccccc
                ************************************************************

Parthenogenetic caataaagtttttccggtggtgcaggtcc
Male            caataaagttt------------------
Female          caataaagtttttccggtggtgc------
                ***********
```
