# Supplementary material for: Glutathione S-transferases play a role in the detoxification of flumethrin and chlorpyrifos in Haemaphysalis longicornis
Source: Parasit Vectors. 2018 Aug 9;11:460. doi: 10.1186/s13071-018-3044-9 (PMC6085608; doi:10.1186/s13071-018-3044-9)
Supplement: Supplementary file 3 — Figure S3. Multiple sequence alignments of the nucleotide sequence of HlGST (a) and HlGST2 (b) of parthenogenetic, male and female ticks. Sequencing was performed using an automated sequencer (ABI PRISM 3100, Genetic Analyzer; Applied Biosystems, Foster City, CA, USA). Multiple sequence alignments of GST genes were done using MAFFT version 7 program (www.mafft.cbrc.jp). Asterisks indicate identity. Start and stop codons are in red. (ZIP 69 kb) [file 13071_2018_3044_MOESM3_ESM.zip › Additional File 3 Figure S3b 20180726.pdf]

b

```
Parthenogenetic gagttgtgctcgatcaagctaccatggcccctgtgctgggatactgggacatccgaggcc
Male            -agttgtgctcgatcaagctaccatggcccctgtgctgggatactgggacatccgaggcc
Female          ----------------agctaccatggcccctgtgctgggatactgggacatccgaggcc
                                ********************************************

Parthenogenetic tttgcgagcccattcgctaccttctggcgcacgctaaagtctcctacgaggataaaaggt
Male            tttgcgagcccattcgctaccttctggcgcacgctaaagtctcctacgaggataaaaggt
Female          tttgcgagcccattcgctaccttctggcgcacgctaaagtctcctacgaggataaaaggt
                ************************************************************

Parthenogenetic acggcttcggcaatggtcccgaacccagccgcgacgagtggttggccgacaagtacaagt
Male            acggcttcggcaatggtcccgaacccagccgcgacgagtggttggccgacaagtacaagt
Female          acggcttcggcaatggtcccgaacccagccgcgacgagtggttggccgacaagtacaagt
                ************************************************************

Parthenogenetic tgggtctggacttccccaacgtgccgtactacatcgacggcgacgtcaagctgacgcaga
Male            tgggtctggacttccccaacgtgccgtactacatcgacggcgacgtcaagctgacgcaga
Female          tgggtctggacttccccaacgtgccgtactacatcgacggcgacgtcaagctgacgcaga
                ************************************************************

Parthenogenetic gcatggccatcctgcagtacctcggccgcaagcacggactcgcccccaaggacgaggcca
Male            gcatggccatcctgcagtacctcggccgcaagcacggactcgcccccaaggacgaggcca
Female          gcatggccatcctgcagtacctcggccgcaagcacggactcgcccccaaggacgaggcca
                ************************************************************

Parthenogenetic ctcagctccgcgtcgacgtgctccagctcacggcgttcgacgtgatcatgtgggcagtgc
Male            ctcagctccgcgtcgacgtgctccagctcacggcgttcgacgtgatcatgtgggcagtgc
Female          ctcagctccgcgtcgacgtgctccagctcacggcgttcgacgtgatcatgtgggcagtgc
                ************************************************************

Parthenogenetic gcgtctgctacgaccccgagtacaccgaggagaagcggaagcagttcctggtcgacgtgg
Male            gcgtctgctacgaccccgagtacaccgaggagaagcggaagcagttcctggtcgacgtgg
Female          gcgtctgctacgaccccgagtacaccgaggagaagcggaagcagttcctggtcgacgtgg
                ************************************************************

Parthenogenetic ccgacaagctgaagcagtttgactcgtacctctccaagtatggtcctttcggcgccggca
Male            ccgacaagctgaagcagtttgactcgtacctctccaagtatggtcctttcggcgccggca
Female          ccgacaagctgaagcagtttgactcgtacctctccaagtatggtcctttcggcgccggca
                ************************************************************

Parthenogenetic agtcagccacttacgtcgacttcttgctctacgaggctctccagatcgtgaaaattcttg
Male            agtcagccacttacgtcgacttcttgctctacgaggctctccagatcgtgaaaattcttg
Female          agtcagccacttacgtcgacttcttgctctacgaggctctccagatcgtgaaaattcttg
                ************************************************************

Parthenogenetic gcccaagcacgttccgcaagggctaccctcagctcgaggagtactgccagcgcgttgctg
Male            gcccaagcacgttccgcaagggctaccctcagctcgaggagtactgccagcgcgttgctg
Female          gcccaagcacgttccgcaagggctaccctcagctcgaggagtactgccagcgcgttgctg
                ************************************************************

Parthenogenetic cccttccgggaatgaaggagtatctggcctcggatcgcttcaaggcttggcccatctgga
Male            cccttccgggaatgaaggagtatctggcctcggatcgcttcaaggcttggcccatctgga
Female          cccttccgggaatgaaggagtatctggcctcggatcgcttcaaggcttggcccatctgga
                ************************************************************

Okayama         gcccgtacgcaaaggcgctggcggcgcagcacaagccgcccgctgacgactgctgagcga
Male            gcccgtacgcaaaggcgctggcggcgcagcacaagccgcccgctgacgactgctgagcga
Female          gcccgtacgcaaaggcgctggcggcgcagcacaagccgcccgctgacgactgctgagcga
                ************************************************************

Parthenogenetic tccccgccggccatcgcgcgtcgcaagcgttgaccgcagttctagtttttgatgttgtt
Male            tccccgccggccatcgcgcgtcgcaagcgttgaccgcagttctagtttttgatgttgtt
Female          tccccgccggccatcgcgcgtcgcaagcgttgaccgcagttctagtttttgatgttgtt
                ************************************************************

Parthenogenetic aatctgggatgcgacgctatatacggctgttacatttttcttttcaataaacggttgcgt
Male            aatctgggatgcgacgctatatacggctgttacatttttcttttcaataaacgg------
Female          aatctgggatgcgacgctatatacggctgttacatttttcttttcaataaacggttgcgt
                ******************************************************

Parthenogenetic cgttttgagcgctcttccgacaaaaaaaaa
Male            ------------------------------
Female          cgttttgagcgctcttccgaca--------
```
